# Demonstrating the potential of a novel spider venom-based biopesticide for target-specific control of the small hive beetle, a serious pest of the European honeybee

**DOI:** 10.1007/s10340-019-01143-3

**Published:** 2019-08-03

**Authors:** Michelle E. Powell, Hannah M. Bradish, Min Cao, Rebecca Makinson, Adrian P. Brown, John A. Gatehouse, Elaine C. Fitches

**Affiliations:** 1 Fera Science Ltd, Sand Hutton, York, YO41 1LZ UK; 2 School of Biosciences, University of Durham, Durham, UK

**Keywords:** Small hive beetle (*Aethina tumida*), European honeybee (*Apis mellifera*), Fusion proteins, ω-hexatoxin-Hv1a, Snowdrop lectin (*Galanthus nivalis* agglutinin)

## Abstract

The parasitic small hive beetle (*Aethina tumida*) feeds on pollen, honey and brood of the European honey bee (*Apis mellifera*); establishment in North America and Australia has resulted in severe economic damage to the apiculture industry. We report potential for the “in-hive” use of a novel biopesticide that is toxic to this invasive beetle pest but harmless to honeybees. Constructs encoding the spider venom neurotoxin ω-hexatoxin-Hv1a (Hv1a) linked to the *N*- or *C*-terminus of snowdrop lectin (GNA) were used to produce recombinant Hv1a/GNA and GNA/Hv1a fusion proteins. Both were similarly toxic to beetles by injection (respective LD_50_s 1.5 and 0.9 nmoles/g larvae), whereas no effects on adult honeybee survival were observed at injection doses of > 200 nmoles/g insect. When fed to *A. tumida* larvae, GNA/Hv1a was significantly more effective than Hv1a/GNA (LC_50_s of 0.52 and 1.14 mg/ml diet, respectively), whereas both proteins were similarly toxic to adults. Results suggested that the reduced efficacy of Hv1a/GNA against larvae was attributable to differences in the susceptibility of the fusion proteins to cleavage by gut serine proteases. In laboratory assays, *A. tumida* larval survival was significantly reduced when brood, inoculated with eggs, was treated with GNA/Hv1a.

**Electronic supplementary material:**

The online version of this article (10.1007/s10340-019-01143-3) contains supplementary material, which is available to authorized users.

## Key message

- The small hive beetle is a serious invasive pest of the European honeybee and represents a serious threat to apiculture.
- Current chemical control methods are limited due to their inherent toxicity towards honeybees.
- Here we report the potential use of a recombinant fusion protein, containing a spider venom neuropeptide linked to a plant “carrier” lectin, as an “in-hive” treatment for the control of small hive beetle larvae and adults which has no toxicity towards bees.

## Introduction

The small hive beetle (*Aethina tumida*) is native to sub-Saharan Africa where it is an occasional parasite and scavenger of African (*Apis mellifera scutellata*) and Cape (*Apis mellifera capensis*) honeybee colonies (Lundie 1940; Smith 1953; Roberts 1971; Elzen et al. 2000). Growth in the international trade of honeybees and hive products over the last two decades has resulted in the spread of *A. tumida* into several countries where its establishment has resulted in significant economic impacts upon the apiculture industry (Elzen et al. 1999; Gillespie et al. 2003).

The domesticated honeybee *Apis mellifera*, generally considered to be the most economically valuable crop pollinator worldwide (Klein et al. 2007), is less aggressive than its African counterpart and allows *A. tumida* adults and larvae to feed unhindered on pollen, honey and brood. Beetle invasion causes honey to ferment, combs to be destroyed, and often the rapid structural collapse and abandonment of hives (Spiewok et al. 2007). A further possible threat to the European honeybee is presented by *A. tumida* acting as a vector for deformed wing virus and the bacterial causative agent of American foulbrood (*Paenibacillus larvae*), which is currently believed to be the most widespread and destructive disease of bee brood (Eyer et al. 2009; Schäfer et al. 2010). Although the effects of *A. tumida* have been particularly severe in North America and Australia, the pest has been detected across a wide range of countries, including Canada, Egypt, Mexico, Sudan, Hawaii, Philippines, Portugal and Italy. This invasive pest represents a serious threat to apiculture as well as wild and feral bee populations, particularly in regions where climatic and soil conditions are conducive to establishment (Neumann et al. 2016). Current *A. tumida* chemical control measures used in America include in-hive (coumaphos containing CheckMite + StripsTM) and soil drench (permethrin) applications, although their inherent toxicity to bees makes effective treatment problematic, and none are currently authorized for use in Europe. Currently used mechanical control methods include a range of traps although their effectiveness is limited as no lure has yet been developed that is more attractive to *A. tumida* than honeybees (Neumann et al. 2016). Given the various issues and limitations of current management options for *A. tumida*, alternative control strategies that are compatible with integrated pest management practices are urgently required.

This paper demonstrates that a recombinant fusion protein comprised of the spider venom neurotoxin Hv1a linked to the mannose binding snowdrop lectin (*Galanthus nivalis* agglutinin [GNA]) has potential utility as an in-hive biopesticide to control *A. tumida*. The 37-residue Hv1a peptide belongs to the ω-HXTX-1 family of cysteine knot toxins originally isolated from the venom of the Australian funnel web spider (*Hadronyche versuta*) (Tedford et al. 2004a). Hv1a targets sites within the insect central nervous system by blocking voltage-gated calcium channels (Fletcher et al. 1997; Tedford et al. 2004b; Chong et al. 2007) and has been shown to be toxic by injection into a range of invertebrates including species belonging to the orders Diptera, Orthoptera, Lepidoptera, Homoptera and Arachnida (Atkinson et al. 1998; Bloomquist 2003; Tedford et al. 2004b; Mukherjee et al. 2006; Fitches et al. 2012; Bonning et al. 2014). By contrast, Hv1a is harmless to vertebrates (Fletcher et al. 1997; Chong et al. 2007) and surprisingly harmless to honeybees (Nakasu et al. 2014), making it an ideal candidate for use in the development of a biopesticide for the control of *A. tumida*. Whilst toxic by injection, Hv1a, like many arachnid venom neurotoxins, is typically far less effective when delivered orally to insects (Fletcher et al. 1997). However, delivery to the haemolymph via leaky septate junctions in the midgut epithelium is thought to confer a degree of oral potency in certain species such as the dipteran species *Lucilia cuprina* and *Drosophila melanogaster* (Herzig et al. 2014; Guo et al. 2018). We have previously demonstrated that linkage of Hv1a to GNA greatly enhances the oral efficacy of the neurotoxin by virtue of the stability of GNA to gut proteolysis, and its ability to cross the gut epithelium and deliver Hv1a to the circulatory system, in larvae of the Lepidopteran *Mamestra brassicae* (Fitches et al. 2012). Fusion to GNA has similarly been used to enhance oral efficacy of the venom toxins SFI1 (*Segestria florentina* toxin 1), PI1a (δ-Amaurobitoxin from *Pireneitega luctuosus*) and ButaIT (Red Scorpion toxin from *Buthus tamulus*) towards a range of insects (Fitches et al. 2004, 2010; Yang et al. 2014; Trung et al. 2006). Analogously, a luteovirus coat protein has been successfully used as a carrier to greatly enhance the oral toxicity of Hv1a to a range of aphid pest species (Bonning et al. 2014).

Bioassays of injection and oral toxicity of recombinant Hv1a, GNA and two Hv1a and GNA containing fusion proteins towards *A. tumida* larvae and adults are reported. Differences in oral toxicity of the fusion proteins are examined in relation to susceptibility to cleavage by beetle gut proteases. Survival of *A. tumida* larvae reared on brood sprayed with fusion protein is reported, and injection studies provide further evidence of the inability of Hv1a to target ion channels in adult honeybees. These results demonstrate the potential of fusion proteins containing Hv1a and GNA as specific biopesticides to control *A. tumida.*

## Materials and methods

### Insect cultures

*Aethina tumida* cultures were maintained in darkness at 20 °C, with 65% relative humidity (RH), in the Quarantine Entomology Unit (Fera Science Ltd.). The culture was originally established from wandering larvae imported under three levels of containment supplied by the Plant Protection Research Institute, South Africa. *Mamestra brassicae* (cabbage looper) originally obtained from cultures held at Fera Science Ltd. and were reared at the University of Durham continuously on artificial diet (Fitches et al. 2012) at 22–25 °C; 65% RH under a 16 h:8 h light:dark regime. Honeybee (*Apis mellifera*) adults and pupae were supplied by Fera Science Ltd. Home Apiary was maintained at 34 °C, 65% RH, under darkness during bioassays.

### Recombinant protein production

**Fig. 1:**
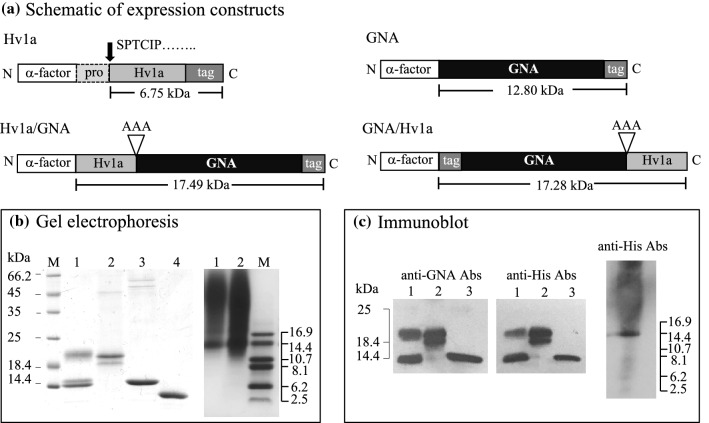
**a** Schematic of constructs encoding recombinant proteins produced in the yeast *P. pastoris* showing predicted molecular masses including tri-alanine linkers. The *α*-factor pre-pro-sequence directs expressed protein to the yeast secretory pathway enabling purification from fermented culture supernatants. Tag denotes the presence of a six-residue histidine sequence that allows recombinant protein purification by nickel affinity chromatography. **b** Separation of purified recombinant proteins by SDS-PAGE gels stained for total protein: left, lane 1 Hv1a/GNA, lane 2 GNA/Hv1a, lane 3 GNA and lane 4 Sigma GNA standard, right, lanes 1 and 2 Hv1a, **c** western analysis of recombinant proteins using anti-GNA and anti-His antibodies, approx. 300 ng total protein loaded in all lanes. Location of protein mass markers run on the same gel are depicted

Constructs encoding for the expression of GNA, GNA/Hv1a and Hv1a/GNA, depicted in Fig. 1a, were generated using previously generated plasmid DNA [Hv1a/GNA accession number JQ8980150 (Fitches et al. 2012)] using conventional PCR, restriction and ligation methods. All constructs include a six-residue histidine tag to enable affinity purification and detection by immunoassay. For Hv1a, a codon-optimized sequence including the predicted 17 residue pro-region (based on Omega-hexatoxin-Ar1d sequence UniprotKB: Accession No. A5A3H3) synthesized by ShinGene Molecular Biotech, Inc. was used as the basis for the generation of the Hv1a expression construct that was cloned into the expression vector pGAPαB in frame with a C-terminal *myc* epitope and histidine tag. Sequence-verified clones in the shuttle vector pGAPZαB were transformed into chemically competent *Pichia pastoris* cells (strain SMD1168H or X33 for GNA) according to Invitrogen protocols. Transformants, selected by plating on zeocin-containing medium (100 µg/ml), were screened for expression in small-scale cultures by Western blotting (using anti-GNA and/or anti-His antibodies) as described previously (Fitches et al. 2012).

For protein production, *P. pastoris* cells expressing recombinant proteins were grown in an Applikon ez-Control laboratory fermenter (7.5 L vessel) as described previously (Fitches et al. 2012). Secreted proteins were separated from cells by centrifugation (30 min at 7500 *g,* 4 *°C*) and clarified by vacuum filtration through 2.7 and 0.7 µM glass fibre filters (Whatman). Recombinant proteins were purified from supernatants by nickel affinity chromatography, dialysed and freeze-dried as described previously (Yang et al. 2014). Protein contents in lyophilized samples were determined from SDS-PAGE gels stained for total proteins with coomassie blue. Quantitation was based on bands corresponding to intact proteins, which were compared to GNA (Sigma) standards by visual inspection, and by capturing an image of the destained gel using a commercial flat-bed scanner; image analysis was carried out with a custom-written software programme (ProQuantify supplied by Rodrigo Guerrero). For Hv1a, which does not resolve well on SDS-PAGE, protein concentrations were determined by BCA assay, using BSA as a standard protein.

### Characterization of recombinant proteins

Proteins were routinely analysed by SDS-PAGE (17.5% acrylamide gels) and western blotting as described previously (Fitches et al. 2012). For the separation of Hv1a polypeptides, SDS-PAGE was carried out at 4 °C using a Tris-Tricine buffer system [15% and 4% (v/v) acrylamide separation and stacking gel, respectively] and gels were run at 100 V.

For *N*-terminal sequencing, Hv1a/GNA and Hv1a proteins were separated by SDS-PAGE and transferred to PVDF membrane (Boehringer GmbH, Germany), stained [coomassie Blue R250 in 40% methanol (v/v)], destained with 50% methanol (v/v), rinsed with distilled water, and excised bands were supplied for *N*-terminal sequencing to Cambridge Biosciences. For LC–MS analysis, proteins in excised gel bands were digested with chymotrypsin or GluC proteases. LC–MS analysis was performed with a Sciex TripleTOF 6600 mass spectrometer coupled to an ekspert™ nanoLC 425 with low micro-gradient flow module (Eksigent) via a DuoSpray source (Sciex). Peptides were separated on a C18 reverse phase column using a gradient of acetonitrile in 0.1% formic acid. MS–MS acquisition used precursor-ion scans (400 to 1600 m/z) of 250 ms followed by CID-fragmentation of up to ten multiply charged ions, with an MS/MS spectrum (m/z 100–1600) acquisition time of 50 ms for each selected ion. MS-Analyst software version 1.7.1 (SCIEX) was used to acquire all MS and MS/MS data, MSConvert (ProteoWizard software suite) was used to generate peak lists and protein identification used Peaks Studio 8.5 in conjunction with a database containing known proteomic-experiment contaminants and the expressed proteins reported here.

### Injection assays

#### *Aethina tumida*

Wandering (10–14 day old) *A. tumida* larvae were injected with 0 to 17.5 µg of protein (in 20 mM sodium phosphate buffer pH 7.4) using a Hamilton micro-syringe. Range-finding experiments were conducted to establish appropriate doses for LD_50_ analysis, after which 4–5 different doses (ten larvae per dose; average wt. 18 mg) of each protein were injected. Larvae were anaesthetized using CO_2_ and 1 μl injected into the third dorsal segment; needles were left in the larvae for 30 s prior to withdrawal to reduce the expulsion of fluid from the wound. Larvae were placed in petri dishes lined with moist filter paper, and survival was monitored daily for 7 days.

#### Mamestra brassicae

Newly eclosed fifth stadium *M. brassicae* larvae (average wt. 60 mg) were anaesthetized with CO_2_ and injected with 5 μl of protein-containing solutions behind the head capsule as described for *A. tumida*. Larvae were placed in plastic pots lined with moist filter paper and containing artificial diet (A.D.), and survival was monitored daily for 7 days.

#### Apis mellifera

Newly emerged *A. mellifera* workers were anaesthetized by cooling on ice and injected using a Hamilton micro-syringe under the fifth abdominal segment with 2 μl containing 10 µg Hv1a, or 40 µg of GNA or GNA/Hv1a (in sodium phosphate buffer). Controls were injected with sodium phosphate buffer alone. Adult honeybees (*n* = 20 per treatment; average wt. 100 mg) were supplied with 50% (w/v) sucrose solution and placed in an environmental chamber (night cycle, 34 °C, 60% R.H.). Survival was monitored daily for 7 days.

### Feeding assays

#### *Aethina tumida* larval “drinking assay”

Five-day-old larvae (*n* = 10 per treatment) were placed in plastic pots containing moist filter paper and two 1.5-ml eppendorf tube lids, each containing protein re-suspended in 75 μl buffer/sucrose (13% w/v) solutions. The liquid diet was changed every 24 h for 72 h, after which larvae were supplied with A.D. [1 ml of 50% (v/w) aqueous honey solution + 2.5 g crushed bee pollen] until they entered the wandering stage (approx. 4 days post liquid feed). Mortality was recorded daily for 7 days.

#### *Aethina tumida* adult “drinking assay”

Adults (1 week old) were transferred into a 50-ml collection chamber containing two 1.5-ml eppendorf tube lids, each containing proteins re-suspended in 75 µl of buffer/sucrose (13% v/w) solution. The diet was changed every 48 h for 6 days, after which adults were supplied with 50% honey solution until day 14.

#### *Aethina tumida* larval “brood assay”

To obtain egg counts, *A. tumida* egg slides were photographed, enlarged on computer and individual eggs counted. Slides containing 290–470 eggs were placed onto honeybee brood (4 cm × 3 cm × depth 3 cm) for 24 h. Slides were then removed, and the brood was sprayed with 1 ml of GNA/Hv1a at a concentration of 5 mg/ml or buffer only as a control. Brood pieces were sprayed (Boots 100 ml travel spray bottle) every 24 h for 72 h after which larvae were supplied with brood ad libitum until they entered the wandering stage when the number of surviving larvae was recorded. During the assay, live and dead larvae displaying a paralytic phenotype were collected for western analysis. Protein extracts were prepared from whole insects (pre-rinsed in dist. water) by re-suspension in sodium phosphate buffer (three larvae in 50 µl) homogenized using a sterile pestle and assayed for protein content using a Coomassie Plus (Bradford) Assay Kit using bovine serum albumin as standard.

### Stability studies

#### In vivo: *Aethina tumida* “larval dipping” assay

Five-day-old larvae (*n* = 10 per treatment) were soaked at room temperature (RT) in buffer/sucrose solutions containing 2.5 mg/ml of GNA/Hv1a or Hv1a/GNA (controls were protein only no larvae), from which 5 μl samples were taken at specified time points for western analysis. Samples were boiled for 10 min, centrifuged (5 min, 14,000*g*) and supernatants stored at − 80 °C until use.

#### In vitro: stability of fusion proteins incubated with *Aethina tumida* larval gut extracts

Feeding-stage larval gut samples were re-suspended in sodium phosphate buffer (10 guts in 200 μl), homogenized using a sterile pestle, centrifuged (5 min at 14,000*g*), and supernatants containing soluble gut proteins were assayed for protein content using a Coomassie Plus (Bradford) Assay Kit using bovine serum albumin as standard. The equivalent of two larval guts (40 µl) was incubated with 75 µg of GNA/Hv1a or Hv1a/GNA at RT and 5 μl samples taken at specified time points. Samples were prepared and stored as described previously.

### Statistical analysis

All survival data were analysed using Kaplan–Meier survival analysis. Median lethal doses (LD_50_ for injections and LC_50_ for feeding experiments) were calculated by plotting log dose versus probit of corrected mortalities. Wandering larvae survival after brood assay experiments was analysed by Chi-square test for significant differences between single values. All statistical tests were carried out using GraphPad Prism software.

## Results

### Recombinant protein production in the yeast *Pichia pastoris*

Four constructs (Fig. 1a), all of which contain a six-residue histidine tag to enable single-step affinity purification, were generated for expression of Hv1a/GNA, GNA/Hv1a, Hv1a toxin alone and GNA as recombinant proteins in transformed *P. pastoris* cells, using a benchtop fermentation system to produce sufficient quantities of proteins for bioassays. Analysis of affinity purified Hv1a/GNA, GNA/Hv1a, GNA and Hv1a by gel electrophoresis is presented in Fig. 1b.

For Hv1a/GNA, a major protein of approx. 21 kDa and two closely separated proteins of approx. 14 kDa were present in purified samples (Fig. 1b); all three proteins reacted positively with anti-GNA and anti-His antibodies (Fig. 1c). The 21 kDa protein was confirmed by *N*-terminal sequencing to contain an additional five residues (Glu-Ala-Glu-Ala-Ala, after removal of the yeast pre-pro-sequence) preceding the Hv1a peptide. Additional residues Glu-Ala-Glu-Ala remain in expressed products due to incomplete processing of the alpha factor sequence by yeast dipeptidyl aminopeptidase, and the additional alanine is a consequence of gene insertion via a *Pst I* restriction site. *N*-terminal sequencing of the two 14 kDa proteins confirmed that these did not contain functional toxin, since cleavage of Hv1a/GNA had occurred at residues 25 and 36 of the Hv1a peptide, leaving only a partial toxin sequence fused to GNA. Thus, only the 21 kDa intact Hv1a/GNA protein was considered when assessing active fusion protein content in lyophilized samples.

For GNA/Hv1a, two proteins of approx. 20 and 19 kDa were apparent in purified fractions (Fig. 1b) and both proteins reacted positively with anti-His antibodies (Fig. 1c), confirming that the *N*-terminus is intact in the expressed products. Analysis of gel slices by LC–MS confirmed (as for Hv1a/GNA) the presence of additional *N*-terminal residues (Glu-Ala-Glu-Ala-Ala) preceding the histidine tag sequence. Similarly to Hv1a/GNA, cleavage at residue 25 of the Hv1a peptide was confirmed by LC–MS analyses of the 19 kDa band, showing this is a GNA/Hv1a cleavage product. Predicted and experimentally determined protein sequence data and representative mass spectra are presented in Online Resource 1(a–c). Thus, only the 20 kDa GNA/Hv1a protein was considered when assessing active fusion protein content in lyophilized samples.

Recombinant GNA runs as a single band of approx. 14 kDa on SDS-PAGE gels, close to the predicted mass of 12.80 kDa, and reacts positively with anti-GNA and anti-His antibodies (Fig. 1c). As evident in Fig. 1b, recombinant Hv1a does not separate well by gel electrophoresis, running as a smear on Tris-Tricine gels. This is indicative of poor binding of SDS to Hv1a and heavy glycosylation. The predicted mass of Hv1a following removal of the pro-region is 6.75 kDa. A protein band of approx. 15 kDa that reacts positively with anti-His antibodies (Fig. 1c) is evident in purified fractions, and an *N*-terminal sequence of Ser-Pro-Thr-Cys-Ile-Pro obtained by Edman degradation sequencing verified removal of the pro-region in the expressed product. All recombinant proteins were expressed at levels of 30–100 mg/l in culture supernatants.

### Biological activity of recombinant proteins

#### Injection toxicity

The biological activity of purified proteins was assessed by injection into *A. tumida* and *M. brassicae* larvae, and *A. mellifera* adults. LD_50_ values are presented in Table 1. For beetle and cabbage looper larvae, mortality following injections of Hv1a/GNA or GNA/Hv1a was predominantly observed 24–72 h post-injection. LD_50_s for *A. tumida* larvae were comparable for Hv1a/GNA and GNA/Hv1a (0.9 and 1.5 nmoles/g larvae, respectively), suggesting that the orientation of the Hv1a toxin relative to GNA in the expressed protein product does not significantly affect functionality of the Hv1a peptide. LD_50_s for Hv1a/GNA or GNA/Hv1a against cabbage looper larvae were also similar (18.0 and 21.5 nmoles/g larvae, respectively) but more than tenfold greater than *A. tumida* values.

**Table 1:** LD_50_ values (nmoles/g larvae) calculated from survival of *A. tumida* and *M. brassica* larvae (five doses, *n* = 10 per dose), and survival (%) of adult honeybee (*A. mellifera*; *n* = 20 per treatment) after injection of recombinant proteins

Treatment	*A. tumida* LD_50_ (day 7)	*M. brassica* LD_50_ (day 5)	*A. mellifera* injection doses (nmoles/g adult bee)	*A. mellifera* survival (%)
Hv1a	6.4 (3.3–11.9)	18.1 (16.4–19.8)	148	100
GNA	–	–	312	95
Hv1a/GNA	0.9 (0.7–1.1)	18.0 (15.4–20.9)	228	95
GNA/Hv1a	1.5 (1.5–1.5)	21.5 (19.1–22.2)	231	90
Control	–	–	0	100

Confidence intervals (95%) are provided in parenthesis

Whilst *A. tumida* larvae showed signs of paralysis after injections of Hv1a alone, mortality was not observed until 6 days after treatment and the observed LD_50_ of 6.4 nmoles/g larvae is more than four-fold higher than the LD_50_ values for fusion protein treatments. Mortality of cabbage looper larvae injected with Hv1a alone also occurred over a longer period (5 days post-injection) although LD_50_s for Hv1a alone as compared to the fusion proteins were similar. No mortality was recorded for *A. tumida* or *M. brassicae* larvae following injections of GNA at doses up to 43 and 30 nmoles/insect, respectively.

Honeybee adult survival was not affected by the injection of Hv1a, Hv1a/GNA, GNA/Hv1a, or GNA at doses up to 20-fold higher than those required to cause mortality in *A. tumida* and *M. brassicae* larvae.

#### Oral toxicity of recombinant proteins to *Aethina tumida* larvae and adults

**Fig. 2:**
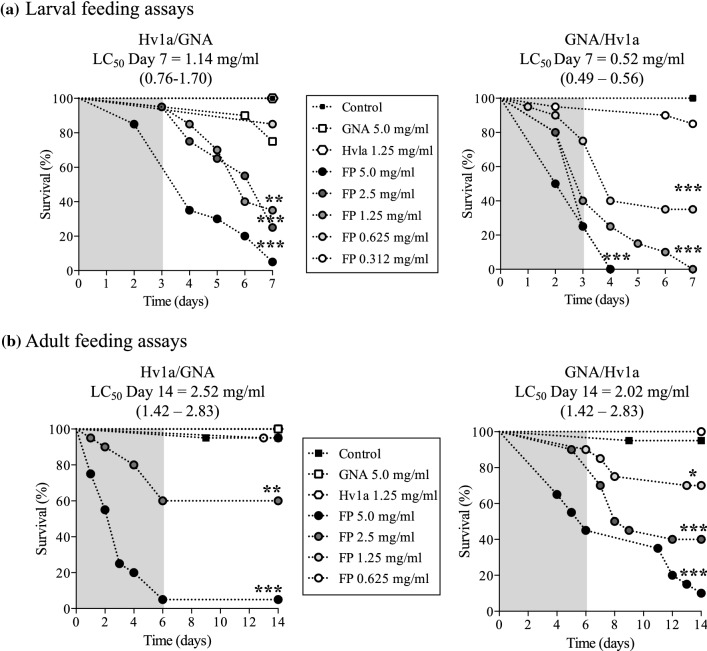
Survival of *A. tumida*: **a** larvae and **b** adults fed on sucrose solutions containing 5 mg/ml GNA, 1.25 mg/ml Hv1a or 0.312–5.0 mg/ml of (i) Hv1a/GNA or (ii) GNA/Hv1a. Controls were fed on sucrose/sodium phosphate buffer. *N* = 20 per treatment. Asterisks depict significant differences to controls (***P < 0.0001, **P < 0.001, *P < 0.05; Mantel–Cox log-rank test). Grey shading depicts fusion protein treatment duration; LD_50_ confidence intervals (95%) for LD_50_ values are provided in parenthesis

The effects of orally delivered fusion proteins on *A. tumida* were evaluated by feeding larvae or adults on solutions containing different concentrations of Hv1a/GNA or GNA/Hv1a, using GNA, Hv1a and no added protein as controls. Solutions were supplied for a limited period (3 days for larvae and 6 days for adults), after which insects were allowed access to control diet. As shown in Fig. 2, significant dose-dependent reductions in survival of larval and adult insects were observed following the ingestion of either Hv1a/GNA or GNA/Hv1a, with mortality > 90% at the highest doses. Insects fed on Hv1a alone showed comparable survival to the control treatments. GNA fed to larvae at the highest concentration of 5 mg/ml caused a reduction in survival of up to 25%, as compared to the control diet-only treatment (*P* < 0.05; Mantel–Cox log-rank test), whereas no reduction in survival was observed for GNA-fed adults.

GNA/Hv1a was more effective as a toxin towards *A. tumida* larvae than Hv1a/GNA. Feeding Hv1a/GNA caused a slower and lesser reduction in survival than GNA/Hv1a, resulting in a significant > twofold higher LC_50_ value of 1.14 mg/ml for Hv1a/GNA as compared to 0.52 mg/ml for GNA/Hv1a. After 2 days of feeding at the higher GNA/Hv1a doses of 5.0 and 2.5 mg/ml, larvae exhibited impaired mobility and a “writhing” phenotype indicative of paralysis; this coincided with the onset of mortality, which even after transfer to control diet, continued to rise, reaching 100% after 7 days. Western analysis of haemolymph samples from larvae fed on fusion protein or GNA provided evidence for the ability of GNA to deliver Hv1a to the circulatory system of *A. tumida* (Online Resource 2).

#### *Aethina tumida* larval “brood assay”

**Fig. 3:**
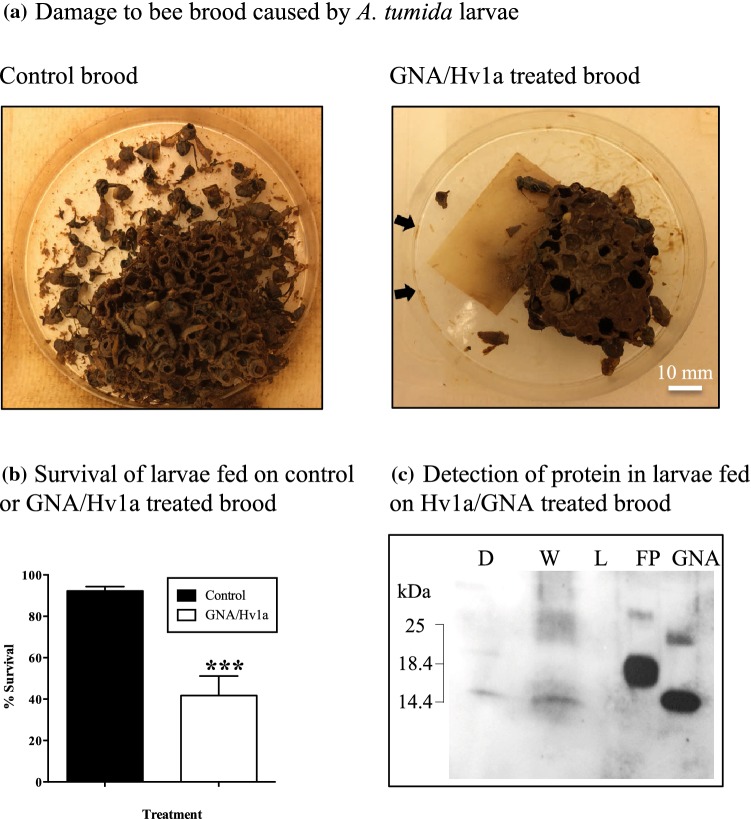
**a** Damage to bee brood caused by feeding *A. tumida* larvae in (i) control brood sprayed with sodium phosphate solution, (ii) brood sprayed with GNA/Hv1a (1 ml of 5 mg/ml FP solution every 24 h for 72 h). Photographic image taken 72 h after the onset of the assay. Arrows indicate dead larvae. **b** Survival of larvae (at the wandering stage) after application of egg slides to bee brood treated with GNA/Hv1a (5 mg/ml in sodium phosphate buffer) or control (buffer only) solution. *N* = 3 per treatment. Asterisks depict significant difference to the control treatment (*P* < 0.0001; Chi-square test). **c** Western analysis (anti-GNA antibodies) of larvae fed on GNA/Hvla-treated bee brood. Extracts (40 µg total protein) prepared from whole dead larvae (D, lane 1); larvae displaying a “writhing” phenotype (W, lane 2) and live (L, lane 3) larvae. Lanes 4 and 5 are, respectively, 100 ng of GNA/Hv1a and recombinant GNA

GNA/Hv1a was selected for use in an experiment designed to mimic an “in-hive” application of a recombinant protein-based biopesticide. *Aethina tumida* egg slides were placed onto honeybee brood which was subsequently sprayed with GNA/Hv1a at a concentration of 5 mg/ml. As shown in Fig. 3a, visual evidence of the ability of *A. tumida* larvae to destroy honeybee brood within 4 days of egg hatch was apparent in the control treatment, whereas fusion protein-treated brood remained relatively intact and dead larvae surrounding the brood piece were clearly visible. Survival of larvae assessed at the wandering stage (i.e. approx. 10 days after the final spray application when larvae cease to feed) was 90–96% for controls, whereas significant levels of mortality (43–76%) were recorded for larvae reared on GNA/Hv1a-treated brood (Fig. 3b; *P *< 0.0001; Chi-square test). Western analysis of larval extracts (Fig. 3c) shows the presence of a single immunoreactive protein band corresponding in mass to GNA, in samples from dead larvae and larvae displaying a paralytic phenotype, but not live larvae. Spraying honeybee brood with protein solution offers the possibility of delivery of the Hv1a toxin to the CNS through contact (via cuticular spiracles) and ingestion, and this result confirmed that protein delivery was responsible for the observed mortality.

#### *Aethina tumida* larval gut proteases and fusion protein stability in vivo and in vitro

**Fig. 4:**
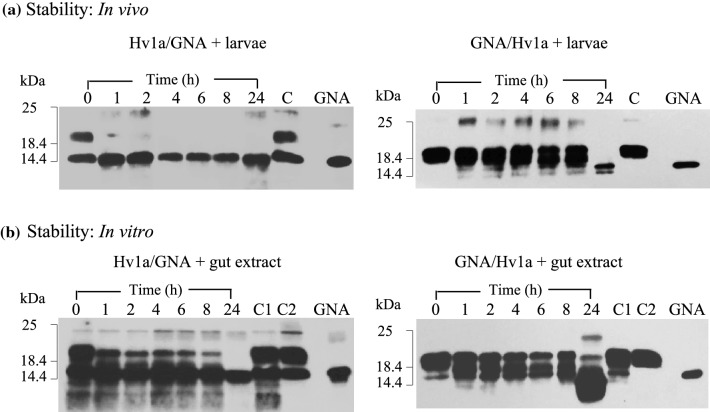
Stability of fusion proteins to degradation by *A. tumida* larval proteases **a** Western blot analysis of solutions [2.5 mg fusion protein (FP)/ml] in which larvae had been immersed for specified time points. C denotes control FP sample alone (no larvae) incubated at RT for 24 h. **b** Western blot analysis of FP samples following incubation with larval gut extracts; 75 µg FP + 40 µl gut extract. For each time point, 300 ng FP loaded. C1 denotes control FP alone, C2 is FP + boiled gut extract, both incubated at RT for 24 h. GNA denotes 100 ng recombinant GNA standard

Two approaches were taken to examine whether variability in the efficacy of Hv1a/GNA and GNA/Hv1a towards larvae was related to differences in the stability of the fusion proteins to proteolytic cleavage. Firstly, larvae were soaked in fusion protein solutions, thereby exposing the proteins to extracellular proteases in the gut, regurgitant and/or frass. As shown in Fig. 4a, differences in the susceptibility of GNA/Hv1a and Hv1a/GNA to proteolytic degradation were evidenced by the disappearance of immunoreactive bands corresponding in mass to intact fusion protein over a sampling period of 24 h. For Hv1a/GNA, samples taken after just 1 h of incubation contained a single immunoreactive band corresponding in mass to GNA alone, indicating that the Hv1a peptide was rapidly cleaved in vivo. By contrast, intact GNA/Hv1a was present in samples incubated with larvae for up to 8 h, with complete cleavage of the Hv1a peptide from the fusion protein to yield GNA alone evident in the 24 h sample. Western analysis of fusion protein samples incubated in the presence of larval gut extracts (Fig. 4b) gave comparable results to in vivo experiments providing further evidence that GNA/Hv1a is more resistant to proteolytic degradation than Hv1a/GNA.

Larval gut proteolytic activity was analysed by zymography, and major protease classes were characterized using protease inhibitors. Results suggested trypsin-like serine proteases that are sensitive to both soybean Kunitz trypsin inhibitor (SKTI) and soybean Bowman–Birk inhibitor (SBBI) are dominant in *A. tumida* larval guts (results not shown). Subsequently, Hv1a/GNA and GNA/Hv1a were both shown to be resistant to cleavage when incubated for 24 h with larval gut extracts in the presence of SKTI, indicating that trypsin proteolysis was responsible for the observed cleavage of fusion proteins.

## Discussion

The purpose of this study was to evaluate the potential use of recombinant fusion proteins comprised of the spider venom peptide neurotoxin Hv1a fused to the carrier protein GNA, as a novel and target-specific method to control *A. tumida*. The specificity of the toxin has been confirmed by injection assays reported here, which show that Hv1a (alone and when fused with GNA) caused high levels of *A. tumida* larval mortality at nanomolar concentrations, whereas no detrimental effects on honeybees were observed. However, feeding assays with larval and adult beetles show that Hv1a alone is ineffective orally and that fusion to the delivery protein GNA is necessary to achieve ingestion toxicity of the fusion proteins. Finally, experiments designed to simulate an “in-hive” application of a fusion protein product demonstrated mortality of *A. tumida* larvae, providing proof of concept for the potential use of fusion protein-based biopesticides to control this invasive pest of the European honey bee.

Hv1a, when tested in native form or as a synthetic or recombinant protein product, has previously been shown to be toxic by injection into a wide range of insects (Fletcher et al. 1997; Tedford et al. 2004b; Bloomquist 2003; Mukherjee et al. 2006). Doses required to induce paralysis and/or mortality are highly variable, ranging from an LD_50_ of 9.4 pmol/g adult fruit fly (*Drosophila melanogaster*) (Guo et al. 2018) to an ED_50_ of 3 nmol/g larvae of the cotton bollworm (*Heliothis armigera*) (Atkinson et al. 1998). Variability in injection efficacy reflects variation in the ability of the toxin to disrupt ion channel function in different insects as well as the use of different sources of peptides and ease of injection. Here, we report an LD_50_ of 6.4 nmol Hv1a/g *A. tumida*, for recombinant Hv1a produced using yeast, demonstrating that the toxin disrupts ion channel function in coleopteran larvae. That Hv1a is a more efficacious towards *A. tumida* as compared to *M. brassicae* is indicated by the three-fold higher injection LD_50_ (18 nmol Hv1a/g insect) obtained for *M. brassicae* larvae. Injection toxicity towards *M. brassicae* larvae was comparable to previous results obtained for recombinant Hv1a produced using bacteria (Fitches et al. 2012), suggesting both expression hosts are capable of producing similar levels of correctly folded and functional toxin.

Injections of GNA/Hv1a or Hv1a/GNA into *M. brassicae* larvae resulted in similar levels of mortality compared to Hv1a alone and were comparable to previously published data for Hv1a/GNA (Fitches et al. 2012). By contrast, *A. tumida* were far more susceptible to injected GNA/Hv1a or Hv1a/GNA where mortality occurred more rapidly and LD_50_s were four-fold lower as compared to Hv1a alone. The reasons for this are not entirely clear but given previous evidence for the binding of GNA and Hv1a/GNA to the central nerve chord of *M. brassicae* larvae, the greater efficacy and more rapid onset of mortality in fusion protein-injected *A. tumida* observed here may reflect the ability of GNA to “deliver” the Hv1a toxin from the circulatory system to the CNS (Fitches et al. 2012). It is also possible that fusion to GNA renders the Hv1a toxin less susceptible to degradation in the haemolymph.

Here, we provide further evidence of the ability of GNA to potentiate the oral toxicity of spider venom peptides as GNA/Hv1a and Hv1a/GNA, when delivered orally, caused dose-dependent reductions in the survival of *A. tumida* larvae and adults, whereas no impact on survival was found for insects fed on Hv1a alone. However, we also show that the orientation of the toxin relative to the carrier GNA has a considerable impact upon efficacy towards *A. tumida*. Larvae and adults were more susceptible to ingested GNA/Hv1a as compared to Hv1a/GNA with differences in efficacy most apparent in larvae, where an LC_50_ of 0.52 mg/ml for GNA/Hv1a was significantly lower (by approx. two-fold) than that obtained for Hv1a/GNA. GNA/Hv1a was subsequently shown to be more resistant to degradation by *A. tumida* gut proteases as compared to Hv1a/GNA. We suggest that the reduced susceptibility of GNA/Hv1a to degradation by gut proteases would enable larger amounts of toxin to be delivered to the haemolymph and CNS, thereby resulting in enhanced toxic effects.

Whilst Hv1a/GNA and GNA/Hv1a contain identical proteolytic cleavage sites, the susceptibility of these sites to proteolysis is presumably determined by how exposed or protected vulnerable sites are to proteases in the tertiary structure of the chimeric protein. Expression of Hv1a/GNA in yeast yields intact fusion protein and two cleavage products; *N*-terminal sequencing confirmed that full-length Hv1a/GNA is susceptible to cleavage during expression at two sites located at the *C*-terminus of Hv1a. That Hv1a/GNA is prone to proteolysis at the *C*-terminus of Hv1a within the insect gut was shown by the rapid appearance of immunoreactive proteins corresponding in mass to GNA alone in samples exposed to *A. tumida* gut proteases (Fig. 4a, b). By comparison, the expression of GNA/Hv1a in yeast yields two products. Western blot and LC–MS peptide mass analysis shows that the larger mass protein is intact fusion protein and the smaller product has been cleaved, as in Hv1a/GNA, at residue 25 at the *C*-terminus of Hv1a peptide. Characterization of recombinantly expressed proteins together with susceptibility to proteolysis in the insect gut collectively suggests that the *C*-terminus of Hv1a is more exposed to both yeast and insect proteases when the peptide is linked to the *N*-, rather than the *C*-terminus of GNA.

In experiments designed to simulate an “in-hive” application of a fusion protein product, we observed significant levels of *A. tumida* larval mortality following the treatment of brood pieces sprayed with GNA/Hv1a over a period of 72 h. In these experiments, it is possible that the fusion protein exerts both contact (via entry through spiracles) and ingestion activity. Indeed, western analysis confirmed that mortality and the observed paralytic phenotype were attributable to ingestion and/or cuticular penetration of the fusion protein. As GNA/Hv1a was only tested at a dose of 5000 ppm, dose–response studies are required to determine the most appropriate practical application rate. Additional experiments are also needed to understand how persistent the effects of GNA/Hv1a are as this would help to determine how much and how often hives should be sprayed to ensure effective control of *A. tumida-*infested hives. It is further anticipated that GNA/Hv1a efficacy and persistence may both be enhanced through formulation. The absence of Hv1a toxicity towards honeybees observed in this study, even when the peptide or GNA fusion proteins were injected at more than 20-fold higher doses than those required to cause significant *A. tumida* larval mortality, is in agreement with results obtained by Nakasu et al. (2014 who reported that Hv1a/GNA shows no contact (adult) or acute oral (adults and larvae) toxicity towards honeybees exposed to 100 μg fusion protein/bee. Nevertheless, chronic toxicity studies are required to establish if there are likely to be any detrimental effects upon honeybee adults and/or larvae exposed to GNA/Hv1a via the repeated spraying of infested hives.

Significant levels of adult *A. tumida* mortality observed following feeding on sucrose solutions containing GNA/Hv1a also suggest the possibility of incorporating the fusion protein in a bait for the control of adults as they enter hives. A number of traps (in and outside hives) are currently used where beetle refuges are filled with control products such as oil, veterinary medicine or a mix of yeast pollen and brood (Neumann et al. 2016). It is likely that GNA/Hv1a would be prone to fairly rapid degradation in the presence of an enzyme-rich brood-based bait rendering it ineffective. Further studies to identify whether GNA/Hv1a could be combined with other bait ingredients or to develop a new fusion protein bait formulation are required. However, efficacious control of adult beetles via trapping is unlikely to be achieved until an effective lure that is more attractive to *A. tumida* than honeybees is developed.

Current measures for *A. tumida* are multifaceted, requiring a combination of all available control methods (mechanical, biological and chemical). The results reported here indicate that fusion protein-based biopesticides could offer the opportunity to reduce reliance upon broad-spectrum chemicals whilst also being compatible with an integrated pest management approach to control this serious invasive pest of honeybees.

## Author contributions

MP executed *A. tumida* and *A. mellifera* experiments assisted by HB. MP, MC and RM produced recombinant proteins, RM carried out *M. brassicae* assays and AB conducted mass spectrophotometry. MP, JG and EF wrote the manuscript.

## Electronic supplementary material

Below is the link to the electronic supplementary material.

Supplementary material 1 (PDF 2785 kb)
