# Supplementary material for: Demonstrating the potential of a novel spider venom-based biopesticide for target-specific control of the small hive beetle, a serious pest of the European honeybee
Source: J Pest Sci (2004). 2019 Aug 3;93(1):391–402. doi: 10.1007/s10340-019-01143-3 (PMC6957549; doi:10.1007/s10340-019-01143-3)
Supplement: Supplementary file 1 — Supplementary material 1 (PDF 2785 kb) [file 10340_2019_1143_MOESM1_ESM.pdf]

Demonstrating the potential of a novel spider venom based biopesticide for target-specific control of the small hive beetle, a serious pest of the European honey bee.

Journal of Pest Science

Michelle E. Powell[1], Hannah M. Bradish[1], Min Cao[2], Rebecca Makinson[2], Adrian P. Brown[2] John A. Gatehouse[2] and Elaine C. Fitches[1,2]

[1]Fera Science Ltd, Sand Hutton, York YO41 1LZ, United Kingdom
[2]School of Biosciences, University of Durham, Durham, United Kingdom
Author for correspondence: Elaine C. Fitches.
E-mail: E.C.Fitches@durham.ac.uk

Online Resource 1

1(a)

ProHv1a
EAEAAAEDTRADLQGGEAAEKVFRR**SPTCIP**SGQPCPYNENCCSQSCTFKENENGNTVKRCDALEQKLISE
EDLNSAVDHHHHHH.

GNA
EAEAAADNILYSGETLSTGEFLNYGSFVFIMQEDCNLVLYDVDKPIWATNTGGLSRSCFLSMQTDGNLVVY
NPSNKPIWASNTGGQNGNYVCILQKDRNVVIYGTDRWATGHHHHHH.

GNA/Hv1a
EAEAAAHHHHHHDNILYSGETLSTGEFLNYGSFVFIMQEDCNLVLYDVDKPIWATNTGGLSRSCFLSMQT
DGNLVVYNPSNKPIWASNTGGQNGNYVCILQKDRNVVIYGTDRWATGAAASPTCIPSGQPCPYNENCCSQ
SCTFKENENGNTVKRCD.

Hv1a/GNA
EAEAAASPTCIPSGQPCPYNENCCSQSCTFK**ENENG**NTVKR**CDAA**ADNILYSGETLSTGEFLNYGSFVFIMQ
EDCNLVLYDVDKPIWATNTGGLSRSCFLSMQTDGNLVVYNPSNKPIWASNTGGQNGNYVCILQKDRNVVI
YGTDRWATGVDHHHHHH.

1(b)

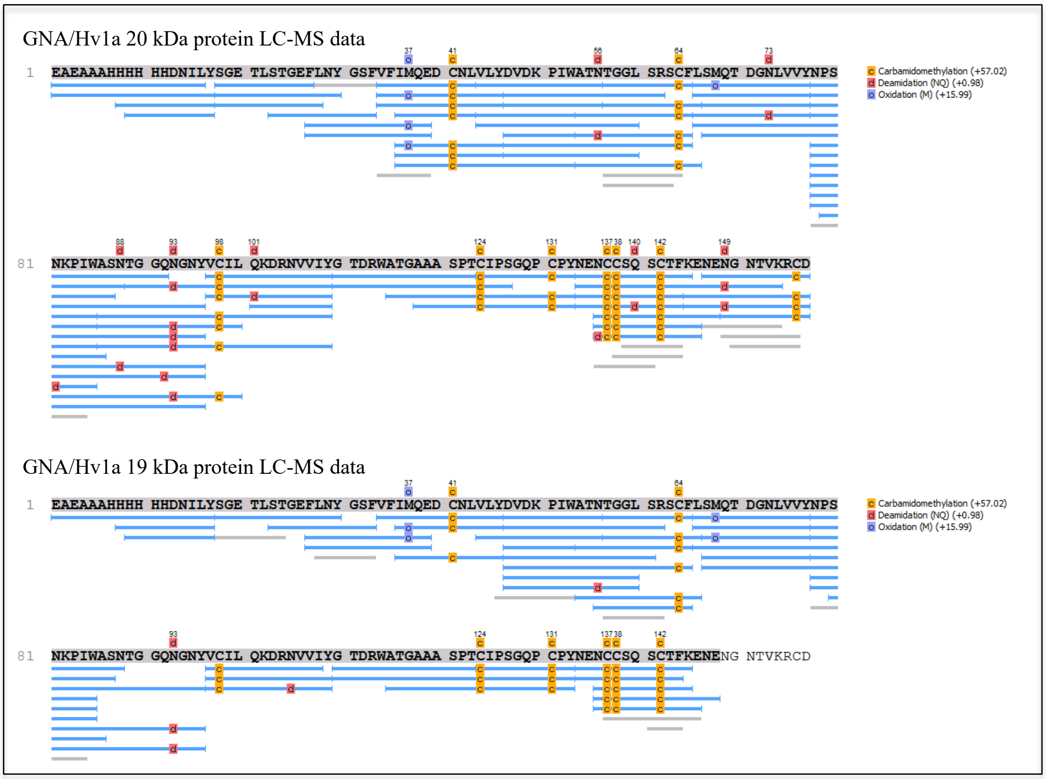

(a) Primary structure of recombinant proteins expressed by transformed *Pichia* pastoris cells. N-terminal residues EAEA depict amino acids present in the yeast pro-sequence that have not been cleaved by the yeast Ste13 gene product (Dipeptidyl aminopeptidase A). The pro-region of Hv1a is depicted in grey, the mature Hv1a sequence in red, the GNA sequence in green, and *myc* epitope (in Hv1a) and histidine tag residues are in blue text. Remaining residues (including the 3-alanine linker in GNA/Hv1a and Hv1a/GNA) are those created during restriction cloning of the expression constructs. Amino acids in bold depict those identified by N-terminal sequencing of gel bands. For GNA/Hv1a, residues in grey highlight depict residues not detected by MS-MS of the 19 kDa cleavage product (see 1b). For Hv1a/GNA, residues in bold with grey highlight depict N-terminal sequences of cleavage products.(b) LC-MS data obtained from digests of the 20 kDa and 19 kDa GNA/Hv1a protein products. Blue bars depict identified peptides and grey bars are sequence tags.

Online Resource 1c : Examples of LC-MS spectra obtained following fragmentation of GNA/Hv1a 20 kDa and 19 kDa proteins.

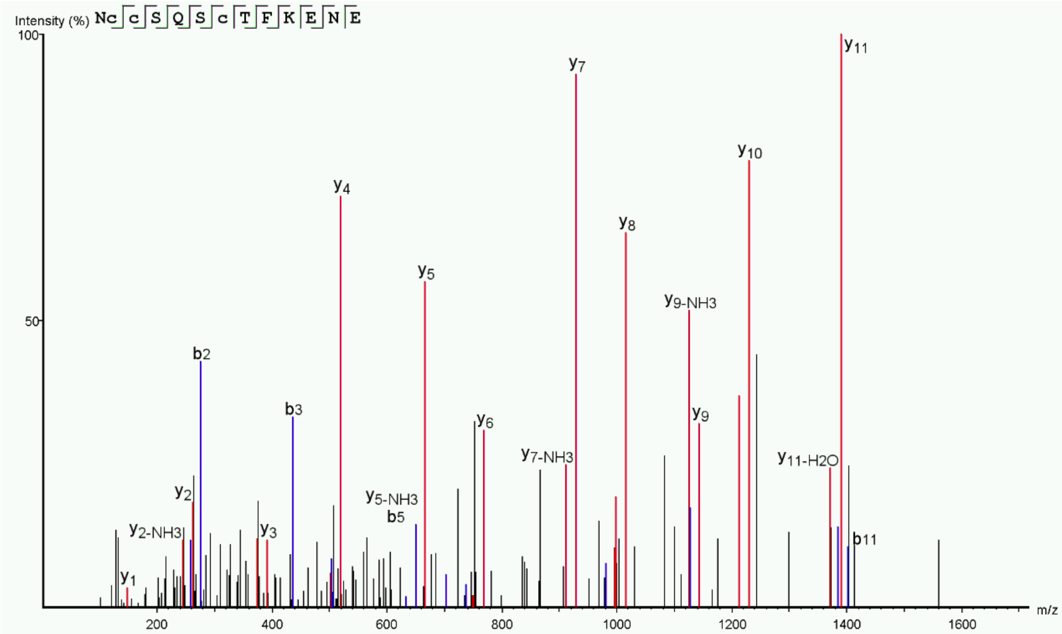

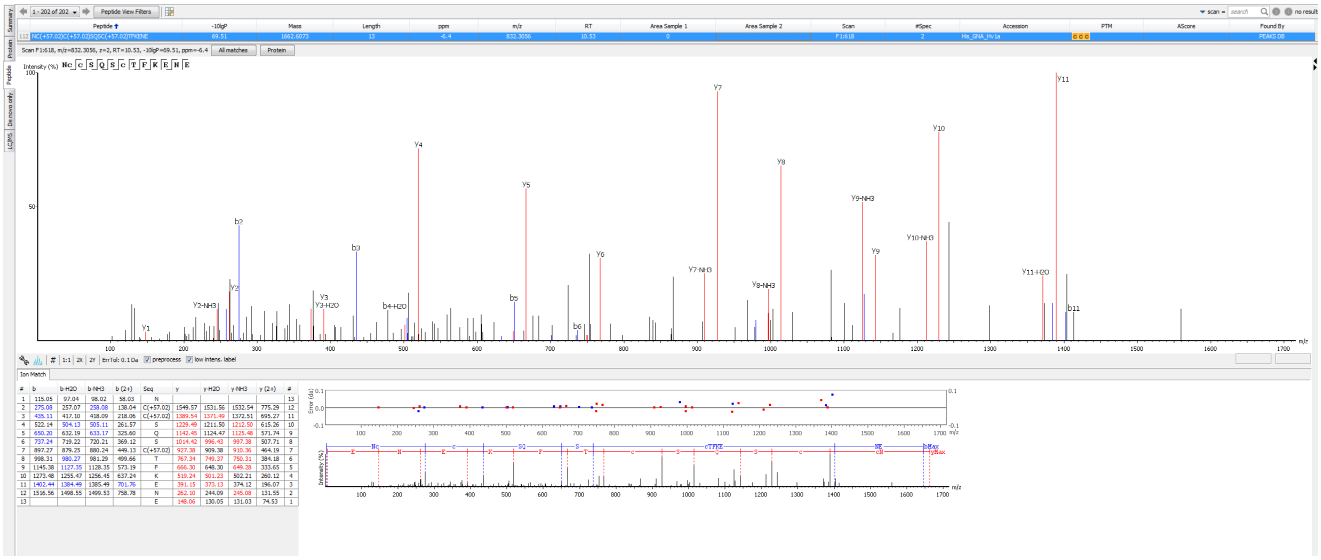

GNA/Hv1a:136-148 NCCSQSCTFKENE (detected in both 19 and 20 kDa GNA/Hv1a protein products)

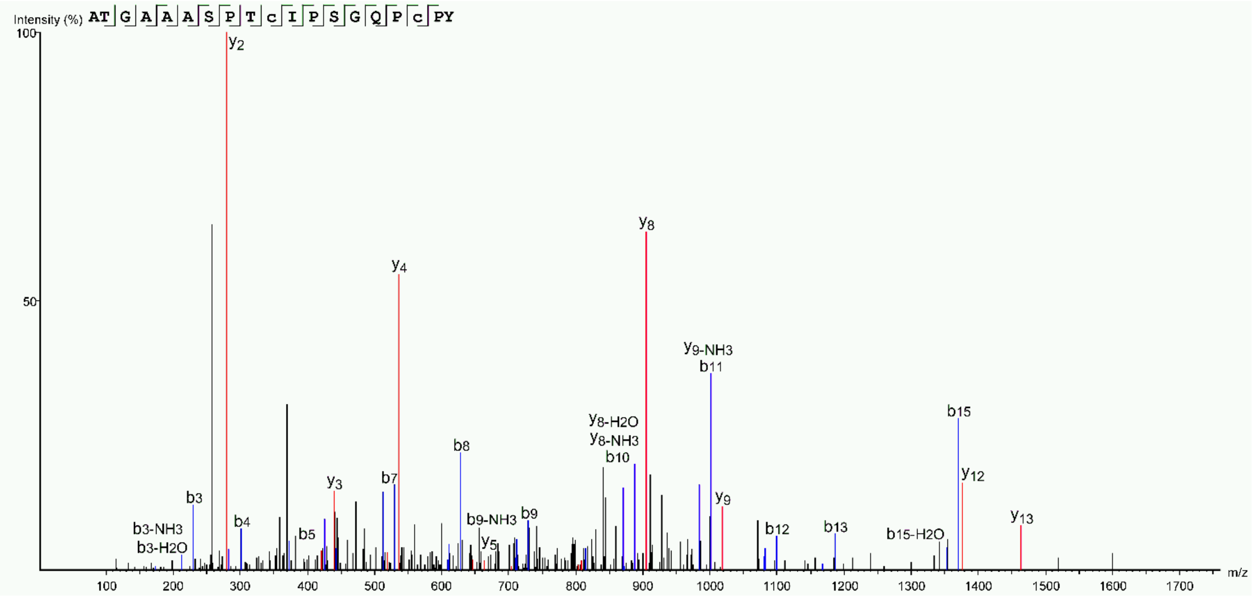

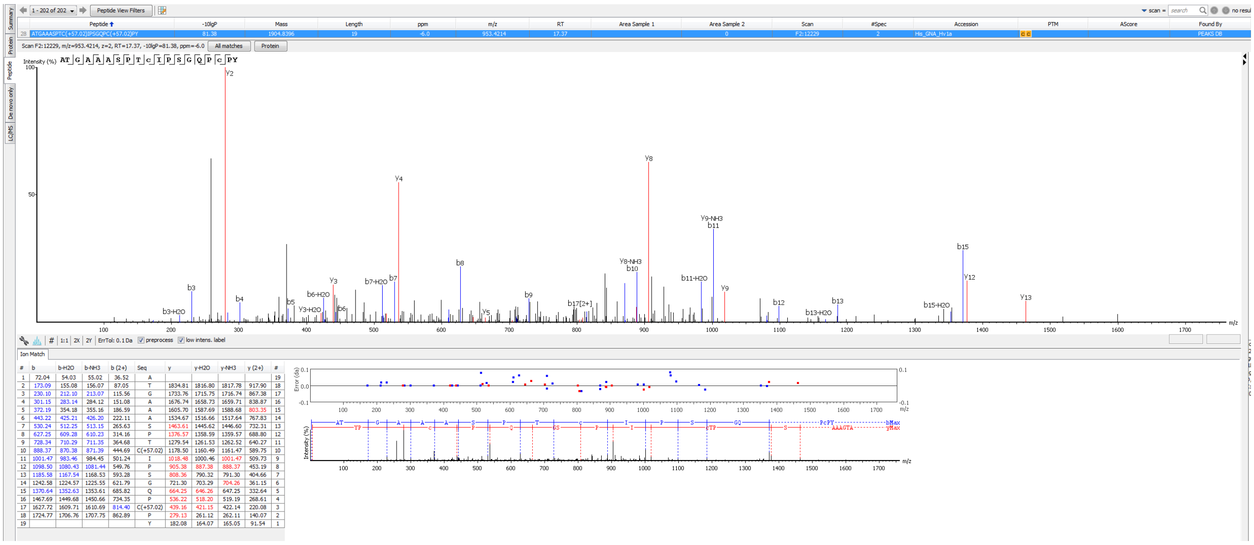

GNA/Hv1a:115-133 ATGAAASPTCIPSGQPCPY (detected in both 19 and 20 kDa GNA/Hv1a protein products)

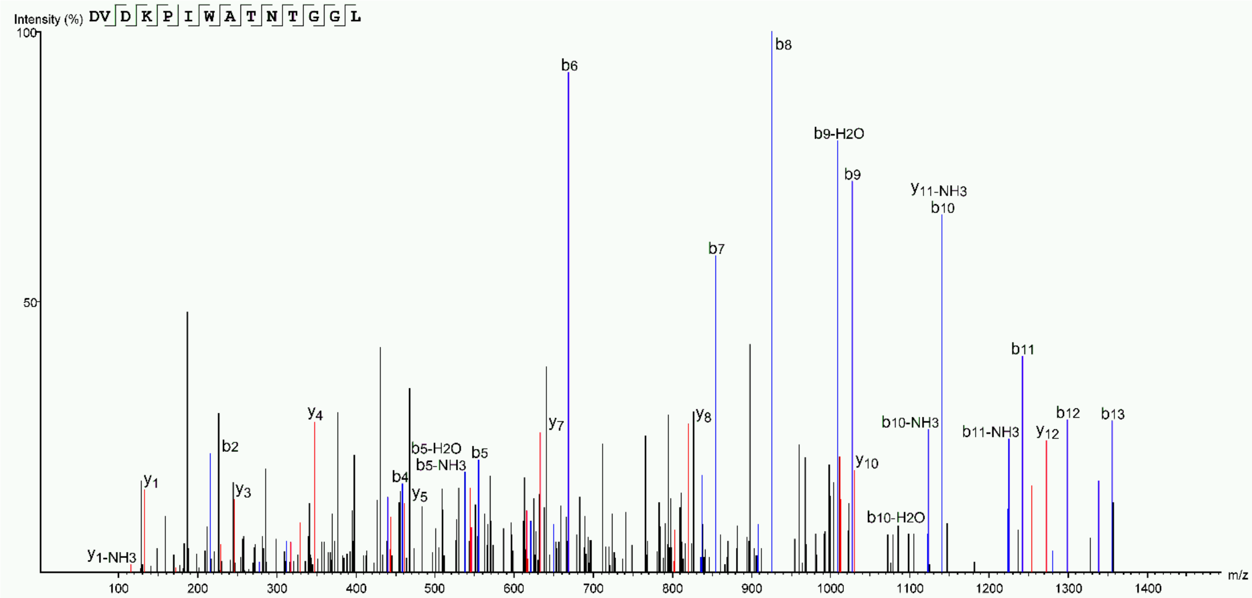

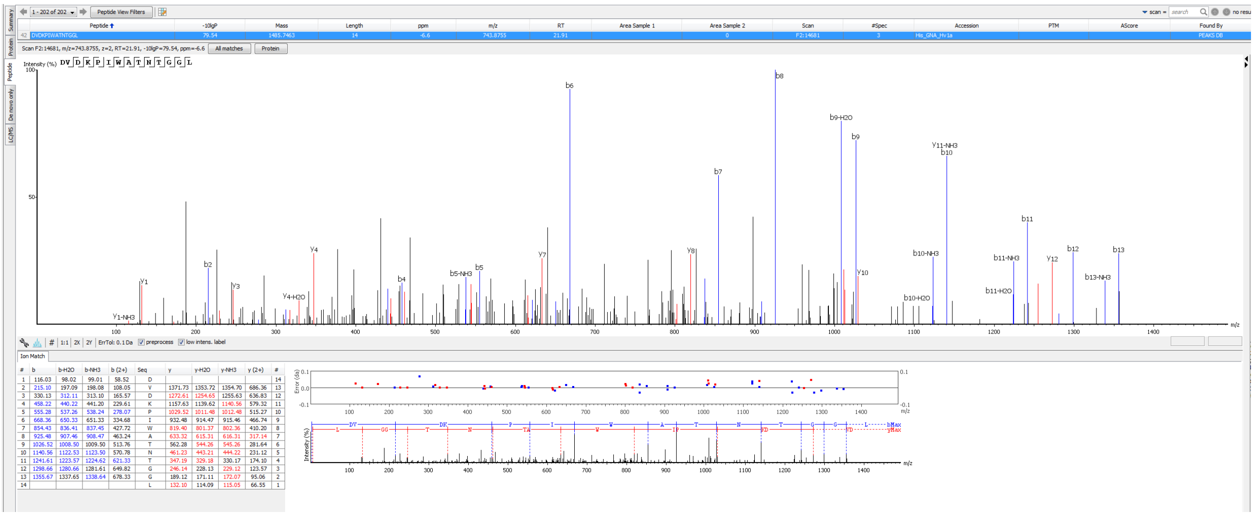

GNA/Hv1a:47-60 DVDKPIWATNTGGL (detected in both 19 and 20 kDa GNA/Hv1a protein products)

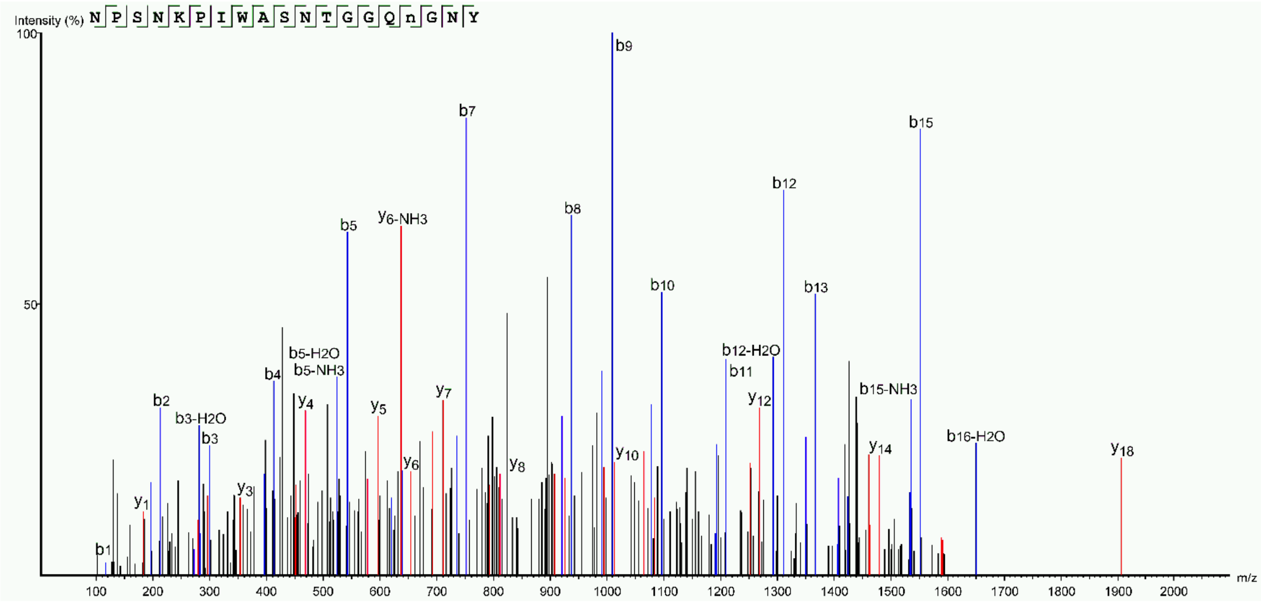

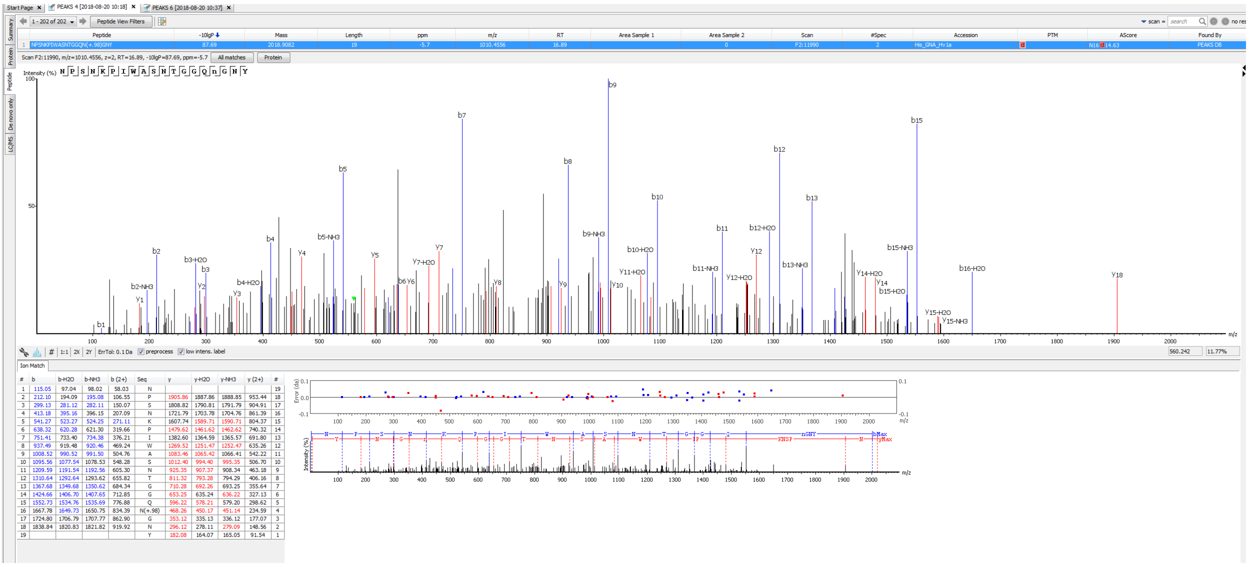

GNA/Hv1a:78-96 NPSNKPIWASNTGGQNGNY (detected in both 19 and 20 kDa GNA/Hv1a protein products)

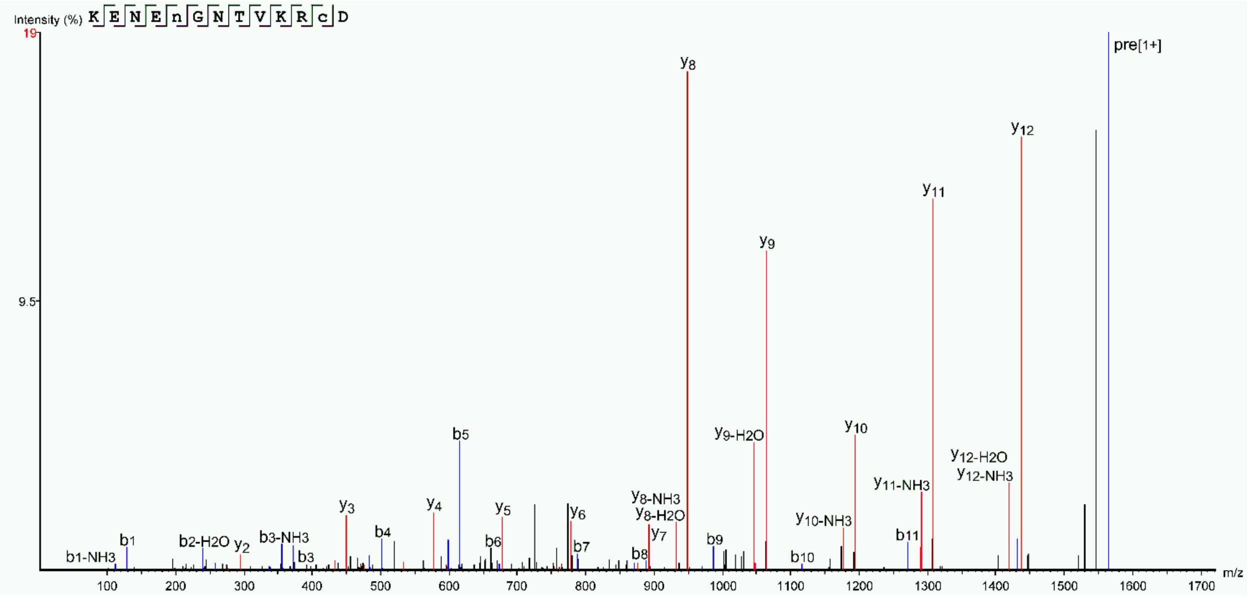

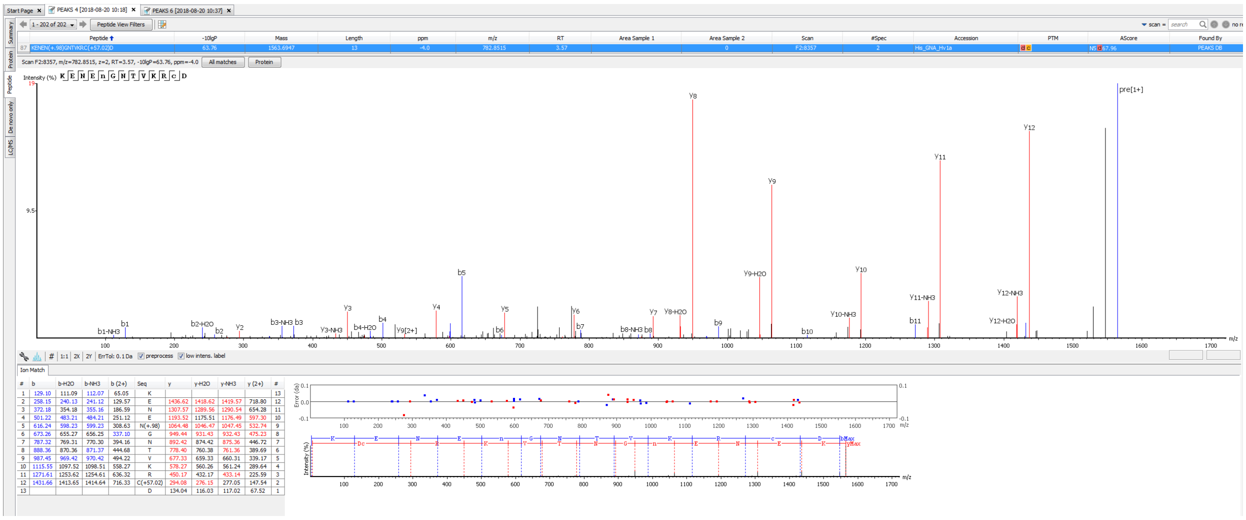

GNA/Hv1a:145-157 KENENGNTVKRCD (detected in 20 kDa GNA/Hv1a protein product)

Online Resource 2

Transport of GNA across the gut epithelium of *A. tumida* larvae.

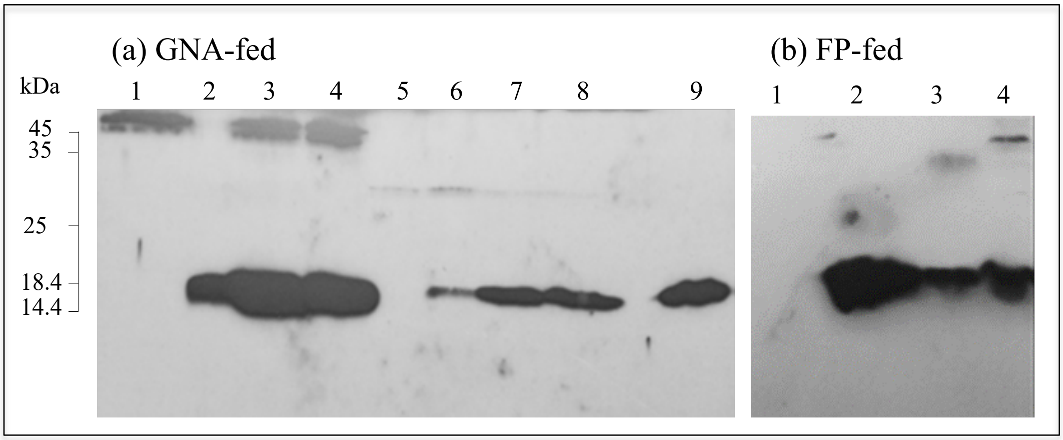

Composite western analysis (anti-GNA antibodies) of 7 day old *A. tumida* larval haemolymph and gut samples after feeding on artificial diet (A.D.) containing 5000 ppm (a) recombinant GNA and (b) GNA/Hv1a fusion protein (FP) for 24 h.
(a) Lanes 1 & 2 show haemolymph (10 µl) of control and GNA fed larvae, respectively; lanes 3 & 4 are replicate pooled gut samples of GNA fed larvae (n=5 per replicate); lane 5 control A.D., lanes 6 to 8 loading of 25, 50 and 100 ng of A.D. containing GNA and lane 9 represents 100 ng recombinant GNA standard.
(b) Lane 1 shows pooled control gut sample (n=5), lane 2 haemolymph (10 µl) from FP-fed larvae, 3 and 4 are gut samples from FP-fed larvae. In all cases 40 µg total gut protein loaded. Mw standards (kDa) based on Ponceau S staining are indicated
